# Cytotoxic and Immunological Effects of Terpinolene and 1‐butyl‐3,4‐methylenedioxybenzene on *Aedes aegypti* Larvae

**DOI:** 10.1002/arch.70212

**Published:** 2026-09-03

**Authors:** Maria Clara da Nóbrega Ferreira Fernandes, Glaucilane dos Santos Cruz, Jaiurte Gomes Martins da Silva, Daniela Maria do Amaral Ferraz Navarro, Jefferson Luiz Princival, Júlio Cesar Ribeiro de Oliveira Farias de Aguiar, Clóvis José Cavalcanti Lapa‐Neto, Emmanuel Viana Pontual, Álvaro Aguiar Coelho Teixeira, Valéria Wanderley Teixeira

**Affiliations:** ^1^ Federal Rural University of Pernambuco (UFRPE) Recife Pernambuco Brazil; ^2^ Federal University of Alagoas (UFAL) Arapiraca Alagoas Brazil; ^3^ Federal University of Pernambuco (UFPE) Recife Pernambuco Brazil; ^4^ Federal University of São João Del Rei São João del Rei Minas Gerais Brazil

**Keywords:** arboviral diseases, dengue, histology, immunohistochemistry, vectors

## Abstract

Interactions between biomolecules and their target sites are fundamental to pest control, and such relationships can either intensify or inhibit their effects. A broader understanding of these complex interactions aids in interpreting toxicological responses, while physiological changes can elucidate insect metabolic pathways. Research involving terpinolene and 1‐butyl‐3,4‐methylenedioxybenzene (BMDB) has demonstrated their insecticidal activity against *Aedes aegypti*. However, the effects of these substances on physiological and immunological parameters require further clarification; furthermore, the binary combination of these compounds may result in more significant and permanent damage. Thus, this study aimed to test the hypothesis that combining different modes of action would intensify histological and immunological alterations critical to insect survival, thereby enabling more effective control. To this end, LC50 values for the individual compounds were obtained from the literature (Silva et al. 2016), and a dose‐response curve was established for the 1:1 combination, revealing an LC50 of 75.69 ± 2.37 ppm. The cytotoxic parameters evaluated included midgut histology, apoptosis, and cell proliferation in L4‐stage larvae, while immunological parameters evaluated included phenoloxidase activity, nitric oxide levels, and oxidative stress markers (TBARS and GST). Histological changes in the midgut were observed in all treatment groups compared to the control; however, more severe histopathological alterations—such as vacuolization, cellular swelling, and loss of the peritrophic matrix—were observed in the combined treatment. Regarding apoptosis, a significant increase was observed only in treatments containing BMDB, whether alone (2.66 ± 0.66) or in combination (3.33 ± 0.33), compared to the control (0.5 ± 0.54). As for cell proliferation (PCNA), larvae in the combined group exhibited a lower rate (0.50 ± 0.11) compared to terpinolene (1.33 ± 0.12) and BMDB (1.40 ± 0.15). Reductions in phenoloxidase activity (1.026 ± 0.061 OD/min/mg) and nitric oxide levels (0.878 ± 0.034 µM/mL) were observed in the combined group. Regarding oxidative stress, there was no statistical difference in GST measurements (*p* > 0.05), whereas TBARS levels decreased in the combined group (2.497 ± 0.184 nmol MDA/mg/protein) compared to the control (3.401 ± 0.327 nmol MDA/mg/protein). Thus, despite exhibiting lower larvicidal activity compared to the isolated compounds, the binary combination caused more severe histopathological and immunological damage that compromises larval survival and recovery capacity, representing a promising strategy for integrated vector management.

## Highlights

- The binary combination of terpinolene and BMDB caused severe midgut damage, including peritrophic matrix loss and cell swelling, in *Aedes aegypti* larvae.
- Only treatments containing BMDB, alone or combined, induced significant apoptosis and reduced phenoloxidase and nitric oxide levels.
- The terpinolene + BMDB association showed the most pronounced cytotoxic and immunological effects, suggesting potential for vector control.

## Graphical abstract

The terpinolene + BMDB association caused severe midgut damage, increased apoptosis, reduced proliferation, and immune suppression in *Ae. aegypti* larvae, indicating that sublethal effects, despite higher LC50, compromise larval recovery and represent a promising strategy for integrated vector management.

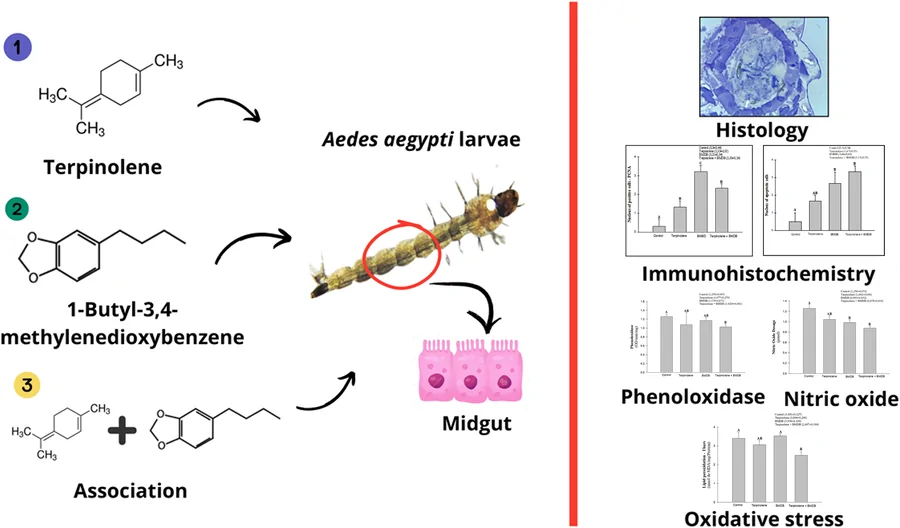

## 1 Introduction

*Aedes aegypti* L. (Diptera: Culicidae) is a primary vector of arboviral diseases such as dengue, Zika, chikungunya, and yellow fever, representing a major public health challenge worldwide (World Health Organization 2024). Its widespread distribution, rapid reproduction, and adaptability to urban environments hinder control through conventional strategies, while resistance to chemical insecticides and environmental concerns underscore the need for alternative, sustainable approaches (Facchinelli et al. 2023; Ebrahim 2025). In this context, botanical compounds have emerged as promising candidates due to their capacity to act on multiple physiological targets.

The disruption of the homeostatic balance in *Ae. aegypti* in response to botanical compounds has been well documented (Bezerra and Pinheiro 2021). This effect arises from interactions among compounds—acting synergistically or additively—at multiple sites of action (Wu et al. 2021). However, most studies have focused on the toxicological and isolated effects, while cytotoxicity remains a relatively underexplored but critical aspect for the development of more effective and environmentally responsible control strategies (Silva et al. 2016; Pereira et al. 2021).

The lipophilic nature of many botanical compounds allows them to penetrate the insect cuticle efficiently, reaching internal physiological systems. Once inside, they may trigger cascades of cellular and immunological alterations, causing lethal or sublethal effects on biology, behavior, development, reproduction, and feeding, as well as inducing repellency and deterrence (Costa et al. 2023; Machado et al. 2023).

Among the primary targets of these compounds is the insect midgut, which has drawn considerable attention from researchers (Cruz et al. 2017; Silva et al. 2019). Damage to the epithelial structure, resulting from apoptosis or necrosis, compromises digestion and nutrient absorption. These disruptions affect the biological cycle and trigger cellular defense responses, including proliferative activity aimed at tissue regeneration to prevent organ dysfunction and, ultimately, organismal failure (Silva et al. 2020).

Following midgut disturbances, immune cascades are activated, in which phenoloxidase and nitric oxide (NO) play crucial roles in epithelial recognition and repair (Cervoni et al. 2017). However, reactive oxygen intermediates produced during this immune response pose a severe threat to cellular structures and DNA, leading to lipid peroxidation, protein oxidation, and lipoprotein modification (Sadekuzzaman et al. 2018).

Considering the complexity of these physiological cascades, substances capable of acting on multiple target sites hold strong potential for reducing *Ae. aegypti* infestation, as they can induce interconnected physiological responses. The cosmopolitan distribution of this vector—driven by globalization, urbanization, and transportation networks—underscores the threat to human health and the limitations of current management strategies (Silva et al. 2020; Vargas et al. 2022).

Among these promising candidates, terpinolene and 1‐butyl‐3,4‐methylenedioxybenzene (BMDB) are plant‐derived compounds with documented insecticidal activity. Terpinolene, a monoterpene, has shown larvicidal effects against mosquito vectors, while BMDB, a phenylpropanoid derivative, can disrupt enzymatic and physiological processes in insects (Silva et al. 2016). Investigating these compounds individually and in combination is particularly relevant, as their interaction may enhance cytotoxic and immunological effects, offering a promising strategy for more effective and environmentally sustainable control of *Ae. aegypti* larvae (Jimenez and Lozano 2022).

Accordingly, the present study aimed to investigate the effects of terpinolene and BMDB, both individually and in combination, on cytotoxic and immunological parameters, including phenoloxidase activity, nitric oxide levels, and oxidative stress). We hypothesized that a binary combination—due to its distinct modes of action—would potentiate cellular damage to the intestinal epithelium and affect immunological parameters in *Ae. aegypti* larvae, thereby reducing the vector population and providing a basis for developing improved, sustainable control strategies.

## 2 Materials and Methods

The research was conducted at the Laboratório de Fisiologia de Insetos (LAFI) and the Laboratório de Estudos Morfológicos em Vertebrados e Invertebrados (LABEMOVI) of the Universidade Federal Rural de Pernambuco (UFRPE), and at the Laboratório de Ecologia Química (LEQ) and the Laboratório de Fisiopatologia Renal of Universidade Federal de Pernambuco (UFPE).

### 2.1 Rearing of the *Aedes Aegypti* Colony

The larvae and adult mosquitoes used in this study originated from the *Ae. aegypti* (Rockefeller strain) colony maintained at the insectary of the Laboratory of Chemical Ecology, Department of Fundamental Chemistry, UFPE. The insectary was kept under controlled conditions of 28°C, 58% relative humidity, and a 10:14 h light–dark photoperiod. Larvae were fed *Whiskas®* cat food pellets, and the water was replaced every other day. Pupae were transferred to cages for adult emergence. Adult mosquitoes were provided with a 10% sucrose solution, and females received a blood meal once per week.

### 2.2 Acquisition and Synthesis of Terpinolene and 1‐butyl‐3,4‐methylenedioxybenzene

The chemical compound terpinolene was purchased from Sigma‐Aldrich® (Brazil) with a purity above 99%. 1‐Butyl‐3,4‐methylenedioxybenzene was synthesized at the Laboratory of Chemical Ecology, Department of Fundamental Chemistry, UFPE, following the methodology described by Pimentel et al. (2022). Briefly, a solution of 4‐bromo‐1,2‐(methylenedioxy) benzene (1.2 mL, 10 mmol) in 30 mL of tetrahydrofuran was cooled to −78°C and maintained at that temperature for 30 min. An n‐butyllithium solution (9.5 mL, 20 mmol) was then added dropwise. The reaction mixture was gradually warmed to 28°C and stirred for 17 h. The reaction was quenched with a saturated ammonium chloride solution, and the product was extracted with ethyl acetate (3 × 15 mL). The organic phase was dried over anhydrous Na_2_SO_4_ and concentrated under reduced pressure using a rotary evaporator.

### 2.3 Establishment of Lethal Concentrations of Compounds and Their Association

The LC_50_ of the compounds terpinolene and 1‐butyl‐3,4‐methylenedioxybenzene used in subsequent experiments were obtained by Silva et al. (2016), with LC_50_ = 31.16 ppm for the compound terpinolene and LC_50_ = 22.1 ppm for the compound 1‐butyl‐3,4‐methylenedioxybenzene. However, that research focused on larvicidal activity and effects at the enzymatic level, leaving a gap regarding potential histological and immunological consequences, a gap this study aims to fill.

To establish the LC_50_ resulting from the association of these compounds, a 1:1 mixture of terpinolene and 1‐butyl‐3,4‐methylenedioxybenzene was made, respectively, according to the methodology described by Cruz et al. (2017). This combination was established based on the premise that the multiple sites of action involved in combining terpenes (terpinolene) and phenylpropanoids (1‐butyl‐3,4‐methylenedioxybenzene) could potentiate deleterious effects, thereby increase mortality and reducing the vector population. The compounds were applied using the methodology described by Silva et al. (2016), where the different concentrations were obtained by diluting the oil and compounds in distilled water and ethanol. Then, twenty larvae in the initial fourth stage (L4) of the Ae. *aegypti* were introduced into 20 mL of each solution in disposable plastic cups (50 mL), 10 cups per concentration, totaling 200 larvae/treatment. The control solution was prepared from distilled water and 0.7 mL of ethanol in a 50 mL volumetric flask. 24 h after installing the experiment, we counted mortality. Larvae were considered dead when they did not respond to the stimulus or when they did not emerge to the surface of the solution (World Health Organization 2005). The results were analyzed using the SAS PROC PROBIT program (SAS Institute 2002). All larval choice experiments were conducted based on a completely randomized design (CRD).

### 2.4 Larvicidal Bioassay of Isolated and Associated Compounds

L4 stage larvae of the *Ae. aegypti* mosquito (Rockefeller strain) was exposed to the LC50 of compounds terpinolene and 1‐butyl‐3,4‐methylenedioxybenzene, both individually and in combination. To this end, twenty immature forms were placed in 20 mL of each solution in disposable plastic cups (50 mL) – 10 cups per concentration, totaling 200 larvae per treatment. The LC50 values were 31.16 for terpinolene, 22.1 for 1‐butyl‐3,4‐methylenedioxybenzene, and 75.69 for the combination. For the negative control treatment, a solution prepared with distilled water and 0.7 mL of ethanol in a 50 mL volumetric flask was used. The larvae remained in the solutions for 24 h. After this period, the surviving larvae were used in subsequent experiments. All concentrations consisted of the substance—either alone or combined – plus water and ethanol.

### 2.5 Histological Analysis of the Midgut

L4 larvae from the control and treated with LC_50_ of the compounds and their association were histologically analyzed 24 h after the experiment was set up and for this purpose, they were fixed in 10% formaldehyde for 24 h and preserved in 70% alcohol. They were then dehydrated in increasing baths of ethyl alcohol (70%− 100%) for 10 min each, soaked in alcohol+historesin (1:1) for 24 h and included in pure historesin (Leica®). Sections measuring 5 μm thick were obtained using a Leica © RM 2035 microtome. The sections were subjected to Toluidine Blue staining techniques for morphological analysis.

### 2.6 Immunohistochemical Analysis of the Midgut

The larvae of *Ae. aegypti* were fixed in 10% buffered formalin for 24 h and preserved in 70% alcohol. The immatures were dehydrated in increasing baths of ethyl alcohol (70%, 80%, 90% and 100%), for 30 min each, diaphanized in xylene, impregnated and embedded in paraffin. Sections of 4 µm thick sections were obtained using a Leica © RM 2035 microtome. Subsequently, the sections were deparaffinized and hydrated and subjected to immunohistochemical analyzes using the TUNEL assay and cell proliferation.

### 2.7 Apoptotic Index in the Midgut

The TUNEL method was used as an indicator of apoptosis. We perform 10 repetitions/treatment. After hydration, the slides were incubated in phosphate‐saline buffer – PBS (pH 7.4 and 0.2 M) at room temperature. For antigen retrieval we used Proteinase K (20 mg/mL). The slides were washed in distilled water and incubated in 3% hydrogen peroxide at room temperature. Sections were washed in PBS and incubated in Equilibration Buffer for 1 h at 4°C. Then, the sections were incubated with the TdT enzyme at 37°C for 1 h in a humid chamber. After this period, the stop solution was applied at room temperature and then the slides were washed in PBS and incubated in anti‐digoxigenin. The slides were rinsed in PBS and the sections were developed with diaminobenzidine chromogenic substrate (DAB, DakoCytomationTM), and counterstained with hematoxylin. The slides were examined under an OLYMPUS BX‐49 light microscope and photographed under a Leica © DM 500 and OLYMPUS BX‐51 photomicroscope. The images were digitized by LAS Leica Image software. The apoptotic index was determined by counting positive nuclei, calculated from the quotient of the number of positive nuclei by the number of total nuclei (positive and negative) and the final value multiplied by 100 (Scudeler et al. 2016). The data were subjected to the normality and homogeneity test, and the means were compared using the Tukey test at 5% probability in the SAS program (SAS Institute 2002).

### 2.8 Cell Proliferation in the Midgut

For this, the PCNA (Cell Proliferation Nuclear Antigen) method was used. We perform 10 repetitions/treatment. After hydration, the slides underwent antigen recovery with Citrate Buffer (pH 6.0) in a microwave oven for 5 min. After this step, when room temperature was reached, the slides were washed in Tris base. To inhibit endogenous peroxidase, the slides were incubated in 3% hydrogen peroxide for 30 min, then washed in Tris base and kept for 30 min in 5% Tris BSA. Incubation in primary specific antibody (PCNA) was carried out in a humid chamber for 18 h. Then, kept in secondary antibody, Histofine ® for 30 min. The slides were rinsed in Tris base and the sections were developed with diaminobenzidine chromogenic substrate (DAB, DakoCytomationTM), and counterstained with hematoxylin. The slides were examined under an OLYMPUS BX‐49 light microscope and photographed under a Leica © DM 500 and OLYMPUS BX‐51 photomicroscope. The images were digitized by LAS Leica Image software. The proliferation index was determined and the result was subjected to the normality and homogeneity test and the means compared using the Tukey test at 5% probability in the SAS program (SAS Institute 2002).

### 2.9 Preparation of Material for Analysis of Immunological Parameters

50 larvae were macerated in 500 µL of 0.1 mM phosphate buffer pH 7.4 in a crucible using a porcelain pestle. The macerate was placed in a 2 mL centrifuge tube and centrifuged for 1 min at 1000 rpm to remove insect exoskeleton fragments. The supernatant was transferred to another 2 mL tube and kept refrigerated at −20°C until analysis. Each treatment consisted of 10 repetitions within 24 h after application.

### 2.10 Phenoloxidase Activity

Analysis of each treatment was carried out with 10 μL of macerate, after 24 h of application. Each treatment consisted of ten repetitions, with 50 larvae being considered in one repetition, totaling 500 individuals per group evaluated. 50 μL duplicates of this mixture were transferred to the microtiter plate. 50 μL of l‐DOPA (L‐ dihydroxyphenylalanine) 4 g/L (Sigma‐Aldrich, St. Louis, MO, USA) was added to each well for enzyme activation. Absorbance was measured at 492 nm using a Biochrom Anthos 2010 microplate reader (Biochrom Ltd., Cambridge, UK) operated by the ADAP software in kinetic mode. Enzyme activity was monitored during the linear phase of the reaction at 60 s intervals for 20 min, according to Faraldo et al. (2005). The data were submitted to ANOVA, and the means were compared using the Tukey test, using the SAS program (SAS Institute 2002).

### 2.11 Nitric Oxide Dosage

The Griess reagent was used (Green et al. 1981), evaluated by the concentration of the nitrite ion (NO^2^^−^). In a pool of 50 μL of the macerate, 70 μL of 1% sulfanilamide in phosphoric acid (H3PO4) (5%) was added. After incubation, 50 μL of the sample (macerated/sulfanilamide) was added another 50 μL of NEED (naphthylethyleneamine dihydrochlorite) at 0.1% in a microtiter plate (Faraldo et al. 2005). Absorbance was measured at 562 nm using a Biochrom Anthos 2010 microplate reader (Biochrom Ltd., Cambridge, UK) operated by the ADP software in endpoint mode. Data were submitted to ANOVA, and means were compared using the Tukey test in SAS software (SAS Institute Inc., Cary, NC, USA).

### 2.12 Analysis of Oxidative Stress

For oxidative stress, lipid peroxidation was assessed by measuring thiobarbituric acid reactive substances (TBARS) acid reactive substances, according to Ohkawa et al. (1979). Fifty larvae from each treatment homogenized in 1.15% KCl + 3 mM EDTA in an ice bath, 1 repetition was considered and 10 repetitions/treatment were performed. Subsequently, we added a reaction medium containing 0.3% thiobarbituric acid, 0.4% SDS and 7.5% acetic acid (pH 3.5), and subsequently the mixture was heated at 95°C for 1 h. The samples were centrifuged, and the supernatant's absorbance was measured at a wavelength of 535 nm. The data obtained were corrected by the protein concentration of the homogenate, measured according to Lowry et al. (1951). Reduced glutathione (GSH) levels were determined by measuring non‐protein sulfhydryl groups, according to the methodology of Sedlak and Lindsay (1968). From the homogenate obtained to evaluate lipid peroxidation, 80–160 mg of tissue were precipitated in a 5% TCA solution. Then, a volume of the supernatant was added to a volume of reaction medium containing 4 mM TRIS, 4 mM EDTA, and 4 mM DTNB at pH 8.9. The reaction was incubated at room temperature for 5 min and the absorbance was measured at 412 nm. The result was corrected for the protein concentration of the homogenate. The analyzes were carried out with 24 after the installation of the experiment. The data were submitted to ANOVA, and the means were compared using the Tukey test, using the SAS program (SAS Institute 2002).

## 3 Results

The results of the larvicidal bioassays, histological and immunohistochemical analyses, and immunological parameters are presented below. Initially, the lethal concentration values for the association of terpinolene and BMDB were determined. Subsequently, the effects of the isolated compounds and their combination on midgut morphology, apoptosis, cell proliferation, phenoloxidase activity, nitric oxide levels, and oxidative stress markers in *Ae. aegypti* larvae are described.

### 3.1 Establishment of Lethal Concentrations of Compounds and Their Association

The toxicity test for the association (Terpinolene + BMDB) revealed a LC_50 value_ of 75.69 ± 2.37 ppm (Table 1).

**Table 1:** Toxicity analysis for the association of terpinolene and 1‐butyl‐3,4‐methylenedioxybenzene compounds.

Compound	At	GL ^b^	CL _50_ (95% CI) ^c, d^ (LCL‐ UCL) ^and^	CL _90_ (95% CI) ^c, d^ (LCL‐ UCL) ^and^
Terpinolene+BMBD	440	3	75.69 ± 2.37^se^ ppm (67.83–83.55)	117.55 ± 2.37 ^se^ ppm (108.35–124.68)

*Note:* The number of larvae used in the test; ^b^degrees of freedom; ^c^lethal concentration and confidence interval; ^d^calculations using Startplus; ^e^minimum and maximum lethal concentrations estimated by the statistical test, followed by the standard error (se).

### 3.2 Histological Analysis of the Midgut

**Figure 1:**
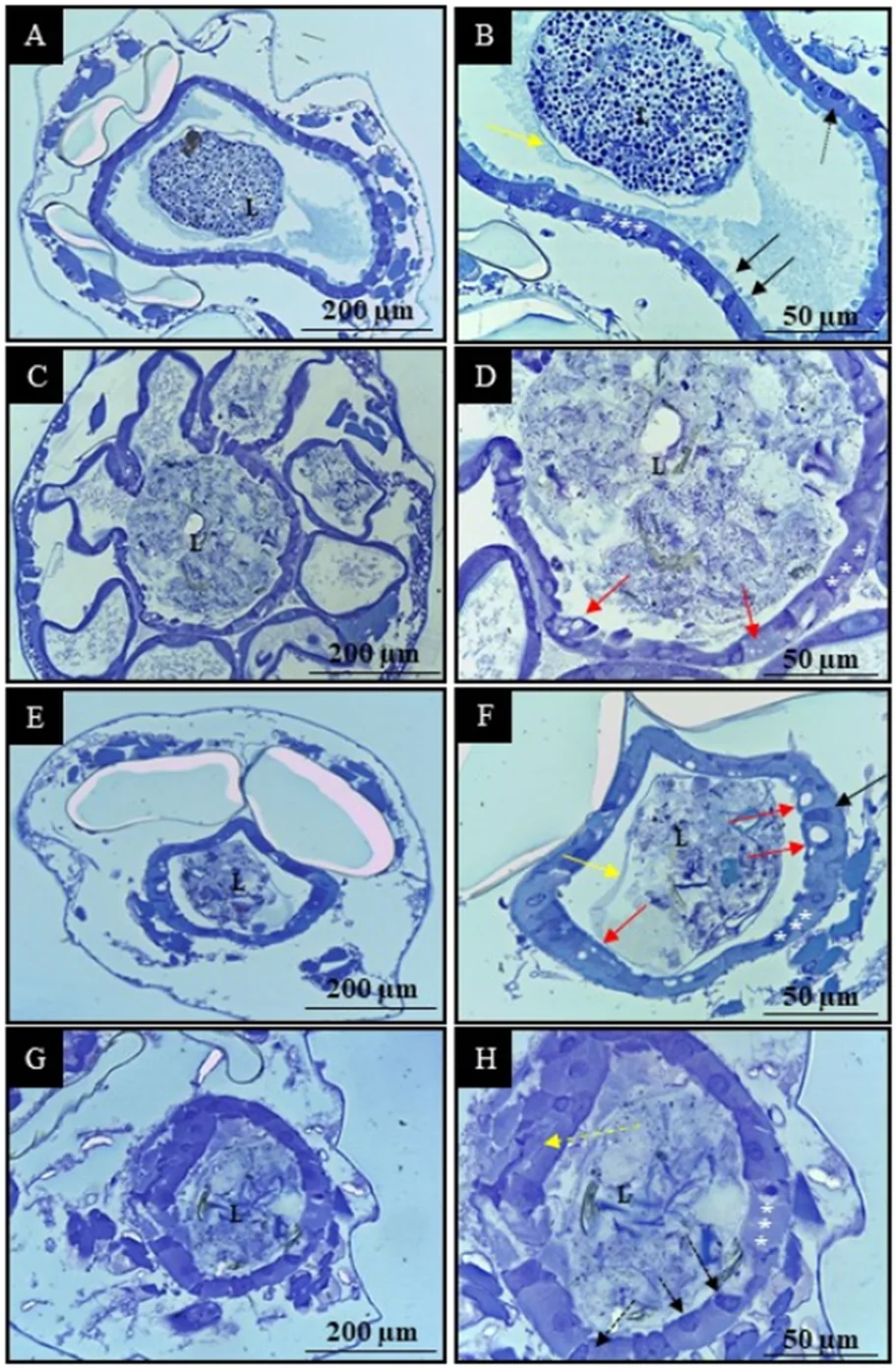
Photomicrograph of the midgut of *Aedes aegypti* larvae at the L4 stage, exposed to the LC50 of the treatments. (A, B) Control, (C, D) Terpinolene, (E, F) BMDB and (G, H) Association (Terpinolene+BMDB). L: lumen, asterisk: columnar epithelium, yellow arrow: peritrophic matrix, red arrow: vacuoles, black arrow: microvilli, dashed black arrow: nests of regenerative cells, dashed yellow arrow: cell swelling.

Larvae from the control treatment had a midgut with a simple layer of epithelium, making it possible to observe two cell types: digestive or columnar cells and regenerative cells. Columnar cells have a spherical and central nucleus, with microvilli‐type specializations in the apical region. Regenerative cells are found at the base of the epithelium, with a pyramidal shape and forming nests. Above the microvilli, it was possible to identify the presence of a peritrophic matrix, separating the food content present in the lumen of the epithelium (Figure 1A,B).

In the midgut of the group treated with terpinolene, as well as in the control group, the presence of simple columnar epithelium was identified. However, it was disorganized and empty. Furthermore, it was not possible to observe the presence of the peritrophic matrix (Figure 1C,D).

In insects treated with the compound 1‐butyl‐3,4‐methylenedioxybenzene, the peritrophic matrix and microvilli were preserved. However, as with terpinolene, the epithelium also appeared disorganized and with the presence of vacuolation, in addition to cells with a swollen appearance. It was also possible to identify nests of regenerative cells (Figure 1E,F).

The histology of the median portion of the intestine of the associated group (terpinolene + BMDB), as well as in insects treated individually with each compound, showed disorganized columnar epithelium with characteristics of cellular swelling, as well as the presence of vacuoles and nests of regenerative cells. The peritrophic matrix was not identified (Figure 1G,H).

### 3.3 Cellular Apoptosis in the Midgut

**Figure 2:**
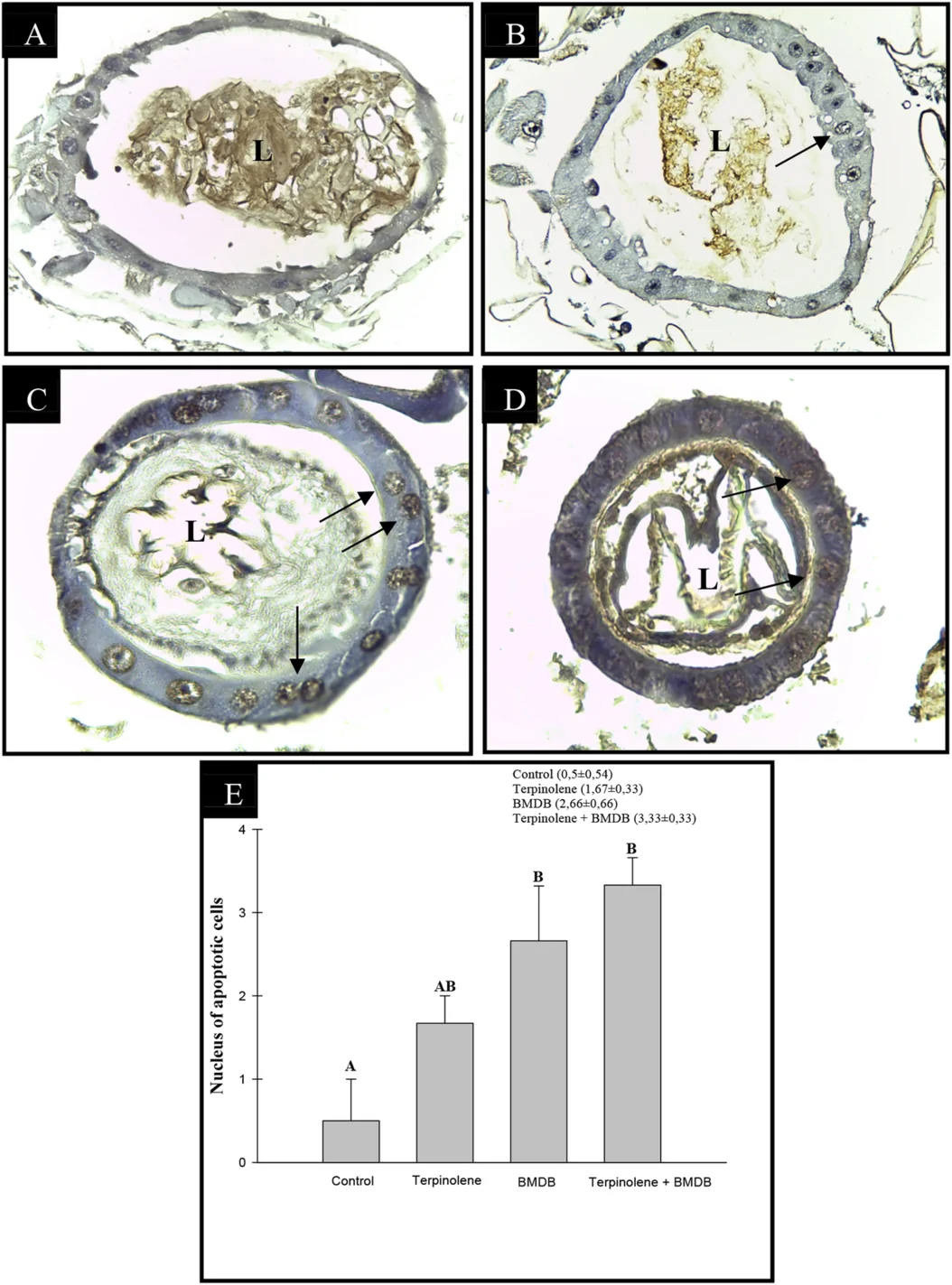
TUNEL test to detect midgut apoptosis of L4 Aedes aegypti larvae (A) Control, (B) Terpinolene, (C) BMDB, (D) Association (Terpinolene+BMDB) and (E) Apoptotic index (mean ± SE). L: Lumen. The presence of apoptosis is indicated by black arrows showing the nuclei with a yellowish color. Bars followed by different letters differ significantly by Tukey's test at the 5% probability level. (F: 44.73; *p* < 0.0001).

The presence of apoptotic nuclei was observed in all treatments. Significant results were expressed by the 1‐butyl‐3,4‐methylenedioxybenzene (2.66 ± 0.66) and association (Terpinolene + BMDB) groups (3.33 ± 0.33) in relation to the control (0.5 ± 0.54). Terpinolene (1.67 ± 0.33), however, did not differ in relation to the control (*p* < 0.001; F = 44.73) (Figure 2).

### 3.4 Cell Proliferation in the Midgut

**Figure 3:**
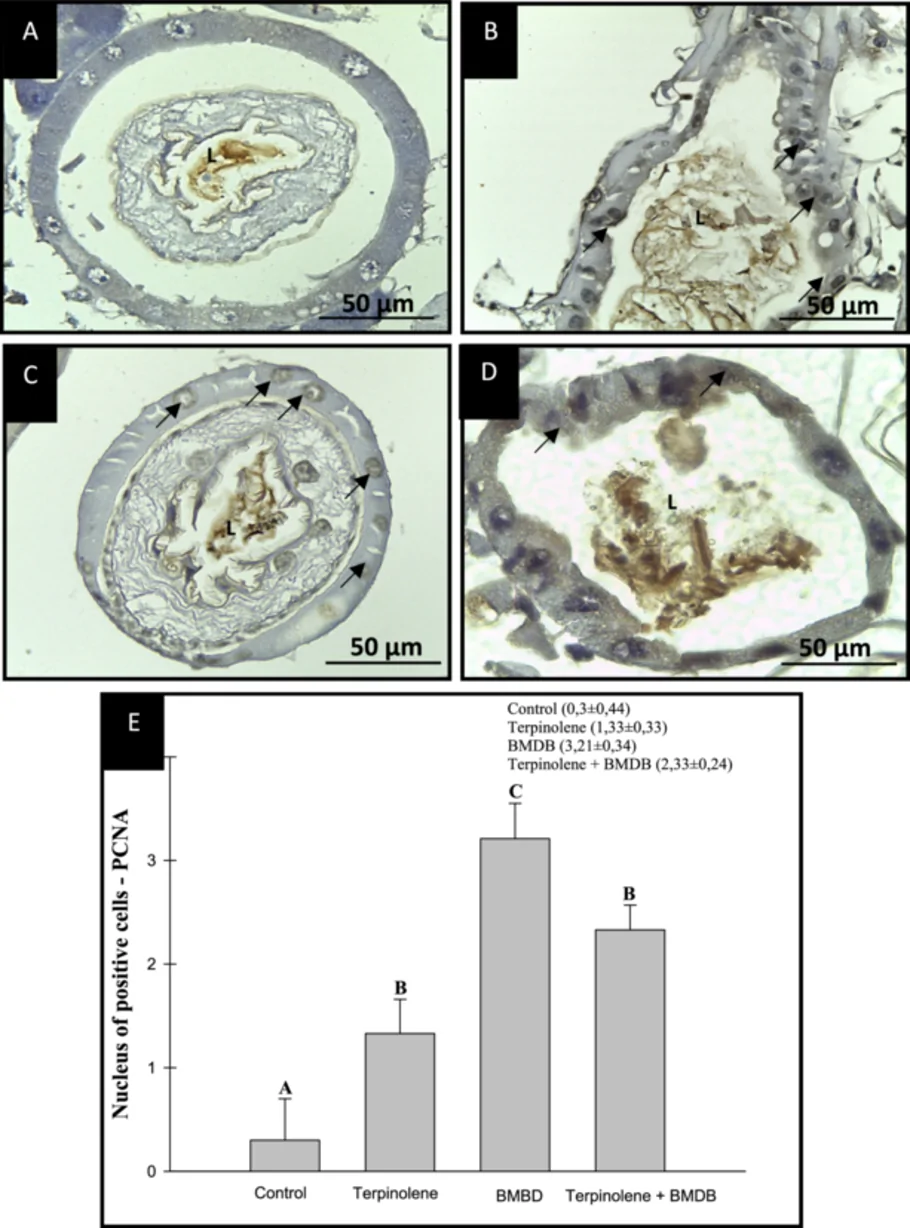
Cell proliferation marker expression. (A) control, (B) Terpinolene (C) BMDB and (D) Association (Terpinolene+BMDB) and (E) Cell proliferation index (mean ± SE). L: Lumen. The presence of proliferation is indicated by arrows showing the nuclei with a yellowish color. Bars followed by different letters differ significantly by Tukey's test at the 5% probability level. (F: 65.67; *p* < 0.0001).

Epithelial cells demonstrated the activation of regeneration mechanisms through the process of cell proliferation in larvae subjected to the compounds. All treatments differed from the control. There was an increase in PCNA markers in larvae treated with terpinolene (1.33 ± 0.12) and 1‐butyl‐3,4‐methylenedioxybenzene (1.40 ± 0.15), in relation to the control. For the association (Terpinolene + BMDB), a reduction was observed in relation to the control (0.50 ± 0.11) (*p* < 0.0001; F = 65.67) (Figure 3).

### 3.5 Phenoloxidase Activity

**Figure 4:**
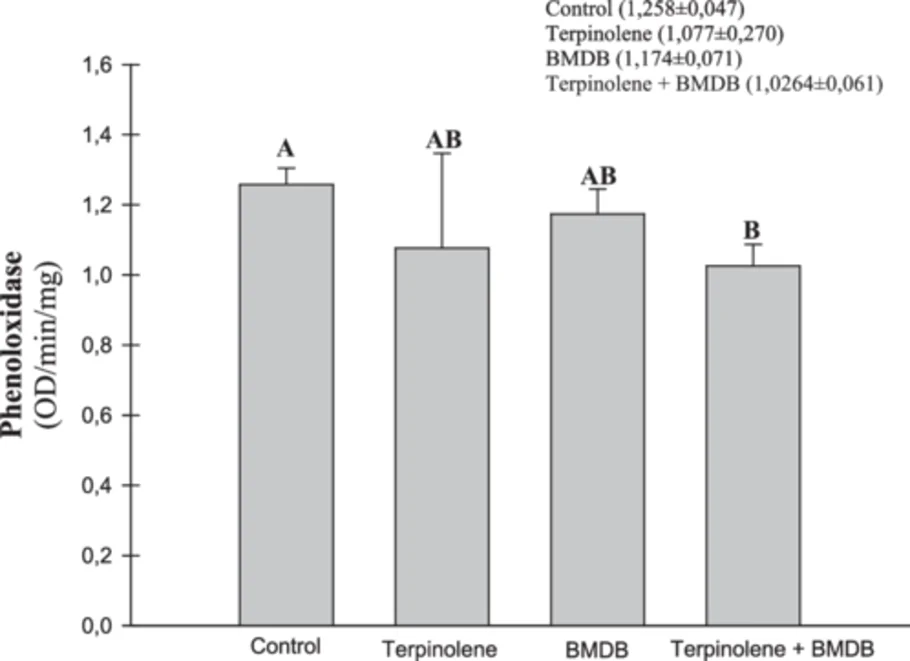
Phenoloxidase enzyme activity (OD/min/mg) of *Aedes aegypti* L4 larvae treated with Terpinolene, BMDB and Association (Terpinolene+BMDB). Bars followed by the same letter do not differ statistically using the Tukey test at the 5% probability level. (F = 3.63; *p* = 0.0218).

When compared to the control, we observed a reduction in the enzymatic activity of phenoloxidase from the associated treatment (Terpinolene + BMDB) (1.0264 ± 0.061 OD/min/mg) after 24 h of exposure. However, we found no difference in relation to the control of the isolated compounds, terpinolene (1.077 ± 0.270 OD/min/mg) and 1‐butyl‐3,4‐methylenedioxybenzene (1.174 ± 0.071 OD/min/mg) (*p* < 0.0218; F = 3.63) (Figure 4).

### 3.6 Nitric Oxide Dosage

**Figure 5:**
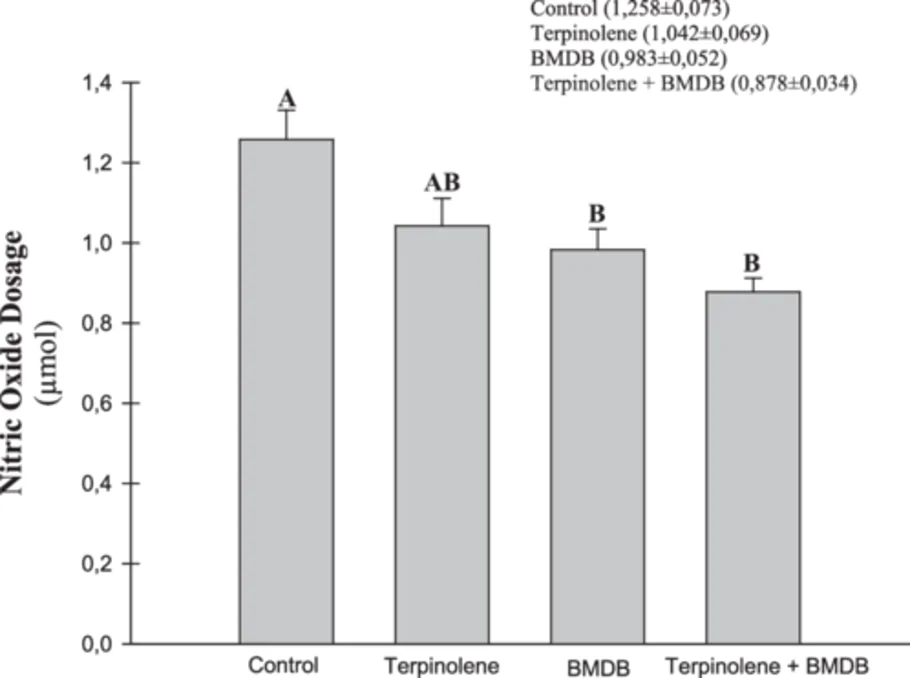
Concentration of Nitric Oxide (µM NO/mL of macerate) of L4 larvae of *Aedes aegypti* treated with Terpinolene, BMDB and Association (Terpinolene+BMDB). Bars followed by the same letter do not differ statistically using the Tukey test at the 5% probability level. (F = 7.37; *p* = 0.0006).

There was a reduction in the dosage of nitric oxide from the isolated treatment with 1‐butyl‐3,4‐methylenedioxybenzene (0.983 ± 0.052 µM/mL) and this associated with terpinolene (0.878 ± 0.034 µM/mL) in relation to the control (1.258 ± 0.073 µM/mL). However, there was no significant difference in terpinolene (1.042 ± 0.069 µM/mL) in relation to the other treatments (*p* < 0.0006, F = 7.37) (Figure 5).

### 3.7 Analysis of Oxidative Stress

**Figure 6:**
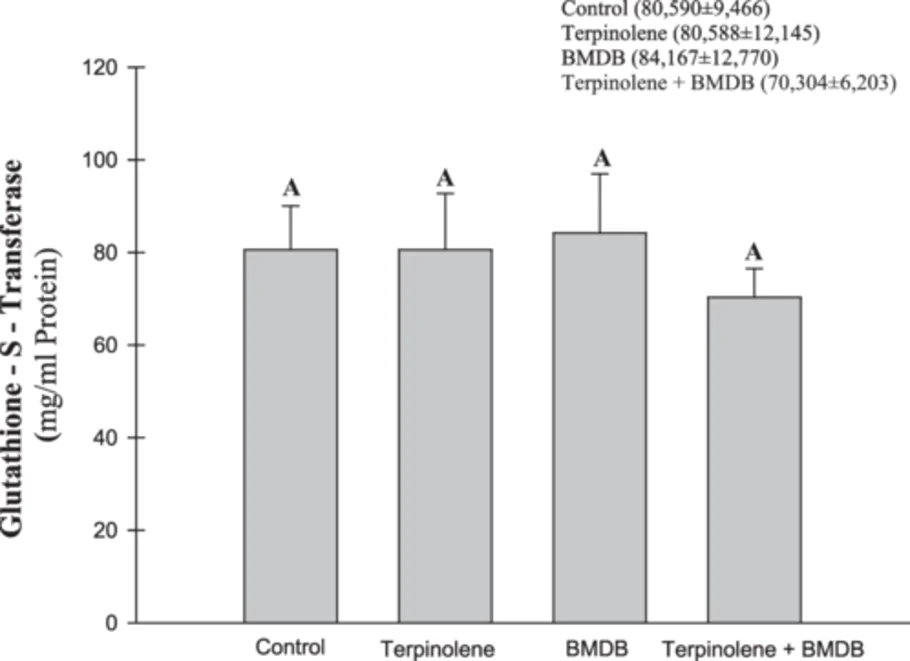
Oxidative stress from the measurement of Glutathione‐S‐Transferase (GST) of L4 larvae of *Aedes aegypti* treated with Terpinolene, BMDB and Association (Terpinolene+BMDB). Bars followed by the same letter do not differ statistically using the Tukey test at the 5% probability level. (F = 3.05; *p* = 0.0482).

There were no differences in the measurement levels of Glutathione‐S‐Transferase (GST) (*p* < 0.0695; F = 2.68) (Figure 6).

**Figure 7:**
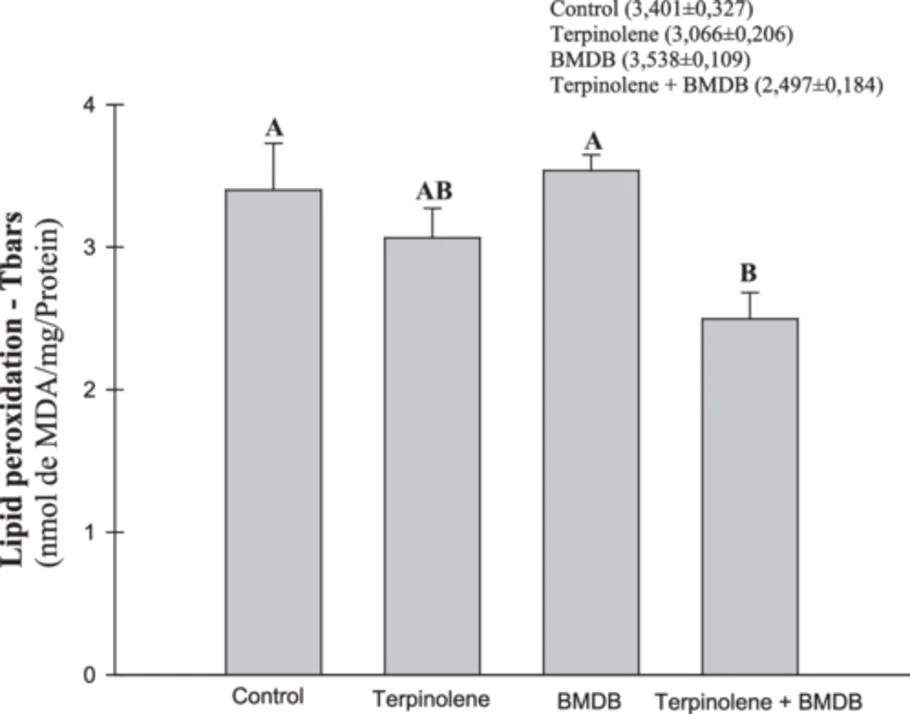
Oxidative stress from the measurement of thiobarbituric acid (TBARS) as an indicator of lipid peroxidation (nmol of MDA/mg/protein) of L4 larvae of *Aedes aegypti* treated with Terpinolene, BMDB and Association (Terpinolene + BMDB). Bars followed by the same letter do not differ statistically using the Tukey test at the 5% probability level. (F = 4.66; *p* = 0.0105).

The reduction in oxidative stress, quantified by TBARS, indicating lipid peroxidation in samples of larvae treated after 24 h of incubation with the associated compounds (2.497 ± 0.184 nmol of MDA/mg/protein) was observed in relation to the control (3.401 ± 0.327 nmol of MDA/mg/protein) and isolated 1‐butyl‐3,4‐methylenedioxybenzene (3.538 ± 0.109 nmol of MDA/mg/protein). Treatment with terpinolene (3.066 ± 0.206 nmol of MDA/mg/protein) did not differ from the control and other groups (*p* < 0.0105; F = 4.66) (Figure 7).

## 4 Discussion

The difference in toxicity results may be correlated to the chemical constitution of each compound. As it is a phenylpropanoid, 1‐butyl‐3,4‐methylenedioxybenzene (BMDB) has greater larvicidal activity against *Ae. aegypti* than terpenoids, such as terpinolene (monoterpene), thus requiring lower concentrations for a more satisfactory toxicological result (Morais et al. 2007). When associated, the compounds required higher concentrations to achieve effective mortality, this fact is probably due to a possible antagonistic action between the mixture (Lima et al. 2019), however, despite this, cellular and immunological responses were more pronounced for insects from this treatment, highlighting the harmful and cytotoxic character that these biomolecules, when associated, played on the vector.

The bioactive compounds present in essential oils directly affect physiological systems and induce a series of cellular and immunological changes capable of causing lethal or sublethal damage to the organism's biology, behavior, development, reproduction, and feeding, while simultaneously exerting repellent and inhibitory effects on the target pest (Costa et al. 2023; Machado et al. 2023). According to Isman (1997), these responses are intensified when an interaction occurs between multiple chemical molecules, and such combinations can also delay the development of resistance mechanisms.

Under normal conditions, the midgut of insect's functions as an extremely important structural immunological barrier against infectious and xenobiotic agents, due to the presence of the peritrophic matrix (Gullan and Cranston 2012). However, with regard to *Ae. aegypti*, the successful systemic viral infection and dissemination of these virulent agents is conditioned on the successful entry and proliferation of these arboviruses in the intestinal epithelium (Ayers et al. 2021). Therefore, intense damage to its structure, in addition to being directly associated with sublethal effects, may be proportional to the increased presence of Reactive Oxygen Species (ROS) and Nitrogen (RNS) in the intracellular environment, resulting from the lack of performance of other immune barriers., may also contribute to preventing the transmission of these diseases to humans (Jabłońska‐Trypuć 2017; Ali et al. 2018; Ayers et al. 2021).

According to Anazetti and Melo (2007), the pattern of cellular morphological and biochemical changes associated with cell death programming includes, among other characteristics, the increase in cytoplasmic vacuoles. On the other hand, cellular swelling is correlated to the necrosis process and occurs due to the increase in cytosolic Na^+^. The increase in cytosolic Ca^2+^ causes activation of phospholipases and proteases, which together with the increase in reactive oxygen species (ROS) induce the rupture of the plasma membrane, activation of proteases with consequent induction of extravasation of cellular contents.

Under natural circumstances, tissue renewal arising from apoptosis or necrosis is a fundamental cellular mechanism for development and is closely correlated with the action of regenerative cells (Caccia et al. 2019). Controlled by the Notch cellular signaling pathway and activated through a series of proteolytic cascades, under adverse conditions the activity of this cell group is indicative of damage that is possibly transient and capable of regeneration (Miller and Zachary 2017). In the present research, positive markers for proliferation denote an attempt to recover the damaged epithelium. However, the reduction or absence of this response, as observed in larvae from the associated treatment, indicates permanent sequelae to the tissue, which may affect the development or lead to the death of the insect (Miranda et al. 2022).

The accumulation of ROS in the system can induce oxidative stress, putting the fundamental structures of cells and tissues at risk, as they result in the loss or incapacity of their functions, through the induction of the expression of apoptotic genes (Camini et al. 2017). Terpenes and phenylpropanoids are natural antioxidants, inhibiting lipid peroxidation by interrupting the peroxidation chain, generating a hydroperoxide and a free radical or by directly buffering free radicals through donating electrons from their hydroxyls, respectively (Yu et al. 2017; Cunha et al. 2018). This fact may explain the absence of increased oxidative stress in all treatments.

Cellular injuries in the organization of the midgut can also activate chemical and enzymatic immunological chain reactions, in addition to negatively affecting other physiological systems dependent on the adequate transformation of food, such as reproduction (Cruz et al. 2017). In this way, aiming to prevent homeostatic collapse, recognition and activity against invading agents can be mediated by the action of phenoloxidase, nitric oxide (NO) and glutathione (GSH) (Silva et al. 2019).

The reduction in phenoloxidase levels in larvae treated with the association and nitric oxide verified with BMDB and association highlights the misfortune caused to the culicidae's immune system. Solidifying these results, Wu et al. (2021) found similar patterns in its levels after exposure *Ae. aegypti* to 2‐thiophenylcoumarin. Phenoloxidase acts to recognize and combat xenobiotic molecules through melanogenesis (González‐Santoyo and Córdoba‐Aguilar 2012). On the other hand, nitric oxide has a considerable impact on non‐specific immunity, through the modulation of anti‐inflammatory reactions, paradoxically, – depending on the cell type and stimulus it receives – this corpuscle can also become cytotoxic and vasodilator to the organism itself (Barbosa et al. 2010).

Therefore, it is concluded that all compounds had adverse effects on *Ae. aegypti*. However, the most severe and possibly permanent damage was observed in larvae subjected to the association of terpinolene with BMDB. This fact is probably due to the interaction between compounds of different classes providing action with multiple active sites. This combination also interferes with the development of resistance, as it is more difficult to detoxify a complex of substances to a single compound (Gutierrez‐Rodriguez et al. 2026). The set of these characteristics associated with the effects mentioned above suggests that this association warrants further investigation as a potential control alternative for Ae. aegypti. However, it is important to emphasize that the present study was conducted under controlled laboratory conditions using a single 1:1 ratio and the LC50 concentration. Therefore, further studies are needed to evaluate the efficacy of this combination at higher concentrations (e.g., LC90) and with different proportions between the compounds. Such investigations would be essential to determine whether the severe sublethal effects observed here translate into effective population control under field conditions, thereby contributing to the development of sustainable strategies for integrated vector management.

## 5 Conclusion

Based on the results obtained, it is inferred that the evaluated treatments induced significant changes in the structural and immunological defenses of *Ae. aegypti*. The most pronounced effects were observed in treatments containing BMDB, particularly when combined with terpinolene. These results suggest that evaluating the potential of a larvicidal agent requires considering physiological changes alongside mortality, as sublethal effects can compromise the individuals' performance and recovery capacity, thereby affecting population dynamics.

## Author Contributions

**Maria Clara da Nóbrega Ferreira:** conceptualization, investigation,writing – original draft, methodology, visualization, writing – review and editing. **Glaucilane dos Santos Cruz:** writing – original draft, methodology, writing – review and editing, conceptualization. **Jaiurte Gomes Martins da Silva:** writing – original draft, writing – review and editing, methodology. **Daniela Maria do Amaral Ferraz Navarro:** methodology, writing – review and editing. **Jefferson Luiz Princival:** methodology, writing – review and editing. **Júlio Cesar Ribeiro de Oliveira Farias de Aguiar:** methodology, writing – review and editing. **Clóvis José Cavalcanti Lapa‐Neto:** methodology, writing – review and editing. **Emmanuel Viana Pontual:** methodology, writing – review and editing. **Álvaro Aguiar Coelho Teixeira:** methodology, writing – review and editing, conceptualization, investigation, funding acquisition, writing – original draft. **Valéria Wanderley Teixeira:** conceptualization, investigation, funding acquisition, writing – original draft, methodology, validation, visualization, formal analysis, writing – review and editing, supervision, project administration.

## Conflicts of Interest

The authors declare no conflicts of interest.

## Data Availability

The data that support the findings of this study are available on request from the corresponding author. The data are not publicly available due to privacy or ethical restrictions.
